# Preoperative Strength Training for Clinical Outcomes Before and After Total Knee Arthroplasty: A Systematic Review and Meta-Analysis

**DOI:** 10.3389/fsurg.2022.879593

**Published:** 2022-07-21

**Authors:** Zugui Wu, Yi Wang, Congcong Li, Junyi Li, Weijian Chen, Zixuan Ye, Ziquan Zeng, Kunhao Hong, Yue Zhu, Tao Jiang, Yanyan Lu, Wengang Liu, Xuemeng Xu

**Affiliations:** ^1^ The Fifth Clinical College of Guangzhou University of Chinese Medicine, Guangzhou, China; ^2^ Guangdong Provincial Second Hospital of Traditional Chinese Medicine, Guangzhou, China; ^3^ Baishui Health Center, Qujing, China; ^4^ Luoyang Orthopedic Hospital Of Henan Province (Orthopedic Hospital of Henan Province), Zhengzhou, China

**Keywords:** preoperative, strength training, rehabilitation, total knee arthroplasty, systematic review

## Abstract

**Background:**

There is an increasing interest in preoperative strength training for promoting post-operative rehabilitation, but the effectiveness of preoperative strength training for clinical outcomes after total knee arthroplasty (TKA) remains controversial.

**Objective:**

This study aims to systematically evaluate the effect of preoperative strength training on clinical outcomes before and after TKA.

**Methods:**

We systematically searched PubMed, Cochrane Library, Web of Science, and EMBASE databases from the inception to November 17, 2021. The meta-analysis was performed to evaluate the effects of preoperative strength training on clinical outcomes before and after TKA.

**Results:**

Seven randomized controlled trials (RCTs) were included (*n* = 306). Immediately before TKA, the pooled results showed significant improvements in pain, knee function, functional ability, stiffness, and physical function in the strength training group compared with the control group, but not in strength (quadriceps), ROM, and WOMAC (total). Compared with the control group, the results indicated strength training had a statistically significant improvement in post-operative knee function, ROM, and functional ability at less than 1 month and 3 months, and had a statistically significant improvement in post-operative strength (quadriceps), stiffness, and WOMAC (total) at 3 months, and had a statistically significant improvement in post-operative pain at 6 months. However, the results indicated strength training had no statistically significant improvement in post-operative strength (quadriceps) at less than 1 month, 6, and 12 months, had no statistically significant improvement in post-operative pain at less than 1 month, 3, and 12 months, had no statistically significant improvement in post-operative knee function at 6 and 12 months, and had no statistically significant improvement in post-operative physical function at 3 months.

**Conclusions:**

Preoperative strength training may be beneficial to early rehabilitation after TKA, but the long-term efficacy needs to be further determined. At the same time, more caution should be exercised when interpreting the clinical efficacy of preoperative strength training for TKA.

## INTRODUCTION

Knee osteoarthritis (KOA) is a major cause of pain and chronic disability in the elderly, which significantly reduces the patient’s quality of life (1). With the development of knee osteoarthritis, the structural damage in the knee joint continues to increase (2). TKA is the most effective and standard treatment in the end stage of knee osteoarthritis, which can significantly reduce pain, improve knee function and quality of life, and has reliable long-term efficacy (3, 4). However, it has been reported that postoperative complications such as persistent pain, proprioception, postural stability, and muscle strength are associated with a high incidence, and such injuries may persist for several years, which has a severe impact on the postoperative recovery of patients (5, 6). Therefore, it is of great significance to explore management strategies to reduce complications after TKA for postoperative rehabilitation. Among the complications after TKA, decreased muscle strength is one of the most significant complications. Related studies have reported that after TKA, muscle strength decreased by 60%, and muscle activity limit reached 17%, which further increased the risk of disability (7). In addition, related studies have found that preoperative quadriceps strength is an essential factor in predicting knee function after TKA (8, 9), and muscle strength is significantly related to pain, knee function, proprioception, and balance function (10). Therefore, preoperative enhancement of muscle strength may be important for rehabilitation after TKA.

Many international guidelines recommend strength training as the core management strategy for knee osteoarthritis (11, 12), and this therapy is also suitable for patients waiting for TKA (13). Some studies suggest that it is appropriate to continue training for 6–8 weeks before operation (14). In the appropriate intervention time frame, muscle strength increases as a result of neural adaptation rather than muscle volume (15). In recent years, more and more clinicians have paid attention to preoperative strength training for patients undergoing TKA. Some evidence supports strength training before TKA. These studies have found that preoperative strength training has multiple positive effects on rehabilitation after TKA, such as reducing pain, increasing muscle strength and range of motion, and promoting joint function and physical function recovery (16–21). However, other study have found that preoperative strength training has no practical clinical significance for the postoperative rehabilitation of patients undergoing TKA (22). Therefore, whether preoperative strength training can promote the postoperative rehabilitation of patients undergoing TKA is controversial, which brings confusion to clinicians’ decision-making. This systematic review and meta-analysis aims to evaluate the effectiveness of preoperative strength training for clinical outcomes before and after TKA.

## METHODS

This systematic review and meta-analysis followed the guiding principle of Preferred Reporting Items for Systematic Reviews and Meta-Analyses (PRISMA) (23). The protocol of this study has been registered in PROSPERO (CRD42021271909).

### Eligibility Criteria

The eligibility criteria for this study were determined according to the principles of participants, interventions, comparisons, outcomes, and study design (PICOS) (24).

### Participants

Participants were diagnosed with knee osteoarthritis and were waiting to undergo TKA. There were no restrictions on participants' age, duration of disease, etc. TKA was performed for the first time, and participants who underwent revision surgery were not included in this study. The surgical approach for TKA is limited to an anterior midline skin incision and a medial parapatellar approach.

### Interventions

• Strength training

Strength training mainly includes muscle strength training (resistance training) of the lower limbs, and muscle strength training of other parts were not included in this study. The main purpose of the training is to improve quadriceps strength. There were no restrictions on strength training methods, such as isokinetic, isometric, or other training methods were eligible for inclusion. In addition, there were no restrictions on the frequency, duration, and intensity of strength training.

### Comparisons

• Strength training vs. other treatments
• Strength training + other treatments vs. other treatments

### Outcomes

- Pain. Pain was measured using the visual analog scale (VAS), Knee Injury and Osteoarthritis Outcome Score (KOOS), or Western Ontario and McMasters University Osteoarthritis Index (WOMAC).
- Knee function. Knee function was measured using the Western Ontario and McMasters University Osteoarthritis Index (WOMAC), Knee Injury and Osteoarthritis Outcome Score (KOOS), or Knee Score (KS).
- Strength (quadriceps).
- Knee range of motion (ROM).
- Functional ability. Functional ability was measured using the Stair test, Timed-Up-and-Go (TUG), or 30-second chair stand test (30s-CST).
- Stiffness. Stiffness was measured using the Western Ontario and McMasters University Osteoarthritis Index (WOMAC).
- WOMAC (total).
- Physical function. Physical function was measured using the Function score (FS), or The MOS 36-item Short-Form Health Survey (SF-36).

### Study Design

The study included only randomized controlled trials (RCTs) and was published in English.

### Search Strategies

We systematically searched the PubMed, Cochrane Library, Web of Science, and EMBASE databases from the inception to November 17, 2021. The following strings and MeSH terms were used for the search: “total knee arthroplasty,” “total knee replacement,” “resistance training,” “strengthening exercise,” “strength training,” and “randomized controlled trial.” The specific literature search strategy was in the Supplementary Appendix. Two independent researchers screened all the literature according to the eligibility criteria. All disagreements were negotiated with the third researcher during the literature screening process, and a consensus was reached.

### Data Extraction

We used Microsoft Excel (2016) software to extract the data required for the study. Standardized tables were developed prior to data extraction. Two independent investigators extracted data from each study according to eligibility criteria, including participant characteristics (e.g., age, duration of disease, gender, diagnostic criteria), study characteristics (e.g., publication years, countries, sample size, frequency of intervention, duration of intervention, follow-up time, adverse events), and outcomes. If there are any questions about the study data, we will contact the author for further confirmation.

### Quality Assessment

According to the Cochrane Handbook (version 5.1.0) (25), two researchers assessed the methodological quality of each study. Methodological quality was assessed from the following seven aspects: (I) random sequence generation (selection bias), (II) allocation concealment (selection bias), (III) blinding of participants and personnel (performance bias), (IV) blinding of related outcomes assessment (detection bias), (V) incomplete outcome data (attrition bias), (VI) selective reporting (reporting bias), and (VII) other bias. Methodological quality was rated as low, high, and unclear. Two researchers used the Grades of Recommendations, Assessment, Development, and Evaluation (GRADE) method to assess the overall quality of evidence for each outcome (24). All disagreements were negotiated with the third researcher, and a consensus was reached.

### Statistical Analysis

Revman Manager software (RevMan 5.3, Cochrane Collaboration) and Stata software (version 16.0) were used to analyze all the data, and forest maps were used to display the pooled results visually. The types of outcomes in this study were continuous variables. According to the merging principle, we pooled data using standardized mean differences (SMD) or mean differences (MD) and calculated 95% confidence intervals (CIs). We used the *I*^2^ test to calculate the heterogeneity of the pooled results. When *I*^2^ ≥ 50% was considered to have significant heterogeneity, the random-effects model was used to merge the data; otherwise, the fixed-effects model was used (26, 27). Sensitivity analysis was used to explore the source of heterogeneity between studies and assess whether the results were robust. In addition, we also used Begg's and Egger's tests to assess publication bias. *P* < 0.05 was considered statistically significant.

## RESULTS

### Study Selection

**Figure 1:**
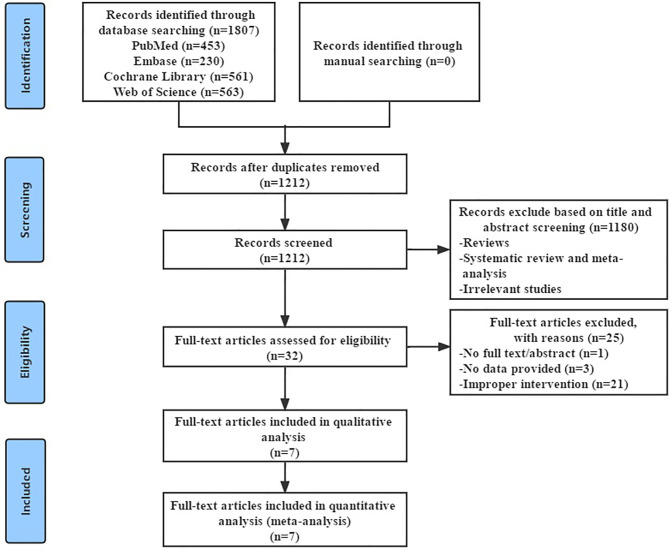
Flowchart of study selection.

The system searched four English databases (PubMed, Cochrane Library, Web of Science, and EMBASE). Preliminarily searched 1,807 possible relevant records and imported them into NoteExpress 3.3.0 software to screen for duplicate literature, titles, and abstracts. After excluding 595 duplicate records, 1,212 records remained. 1,180 records were excluded by reading the title and abstract, and the remaining 32 records need to be downloaded in full text for further confirmation. By reading the full text of the literature, 25 articles that did not meet the inclusion criteria were excluded (e.g., improper intervention, no data provided), and 7 articles remained. After further reading, the 7 articles were confirmed to meet the inclusion criteria and were finally included for systematic review and meta-analysis (16–22) (Figure 1).

### Study Characteristics

Seven studies were included, including 306 participants, and all included studies were randomized controlled trials (RCTs). The basic characteristics of these studies were summarized in Tables 1 and 2. These studies were from the USA (16), Thailand (17), Denmark (18), Spain (19, 21), Bosnia and Herzegovina (20), and the Netherlands (22). Seven studies were published from 2009 to 2021, with sample sizes ranging from 18 to 60. Six studies reported the average age of the participants, whose average age ranged from 63.00 (7.60) to 71.8 (7.5). Only one of the seven studies included used the American College of Rheumatology (ACR) diagnostic criteria (19), and the other six studies were all physician diagnoses. The duration of intervention in these studies ranged from 3 weeks to 20 weeks, and the follow-up time ranged from 1 week to 12 months after surgery. In addition, these studies used different outcomes and assessment methods. Five studies that assessed pain used VAS (16, 19), KOOS (18, 21), and WOMAC subscales (17), respectively. Five studies that assessed knee function used KOOS (18, 21), WOMAC subscales (17, 19), and KS (20), respectively. Six studies assessed strength (quadriceps) (16–19, 21, 22), and four studies assessed ROM (17–19, 21). Five studies that assessed functional ability used the stair test (19, 22), TUG (21), and 30s-CST (16, 18), respectively. Two studies that assessed physical function used SF-36 (19), and FS (20), respectively. Three studies assessed WOMAC(total) (17, 19, 22), and two studies reported stiffness using the WOMAC subscale (17, 19).

**Table 1:** Study characteristics.

References	Diagnosis	Type of operation	Publication years	Country	Study design	Mean age (SD), years		Sample size		Male/Female		Droup out	
						IG	CG	IG	CG	IG	CG	IG	CG
Topp et al. (16)	KOA^a^	TKA	2009	USA	RCT	64.10(7.05)	63.50(6.68)	26	28	17/37	NR		
Tungtrongjit et al. (17)	KOA^a^	TKA	2012	Thailand	RCT	63.00(7.60)	65.90(7.20)	30	30	4/26	6/24	8	
Skoffer et al. (18)	KOA^a^	TKA	2016	Denmark	RCT	70.70(7.30)	70.10(6.40)	30	29	11/19	12/17	6	9
Calatayud et al. (19)	KOA^b^	TKA	2017	Spain	RCT	66.80(4.80)	66.70(3.10)	25	25	4/18	3/19	3	3
Jahic et al. (20)	KOA^a^	TKA	2018	Bosnia and Herzegovina	RCT	NR	10	10	3/7	3/7	0	0	
Domínguez et al. (21)	KOA^a^	TKA	2021	Spain	RCT	70.80(5.40)	70.20(5.60)	24	21	10/14	7/14	5	5
Leeuwen et al. (22)	KOA^a^	TKA	2014	Netherlands	RCT	71.80(7.50)	69.50(7.10)	10	8	7/3	4/4	1	3

*ACR, American College of Rheumatology; IG, Intervention group; CG, Control group; RCT, Randomized controlled trial; NR, Not reported; KOA^a^, physician diagnoses; KOA^b^, American College of Rheumatology (ACR) diagnostic criteria; TKA, Total knee arthroplasty*.

**Table 2:** Intervention characteristics and outcome measures.

References	Intervention characteristics		Main outcomes and results	Follow-up	Adverse events
	Intervention group	Control group			
Topp et al. (16)	Strength training (3 times a week; 20 weeks)	Usual care	1. Pain (VAS) 2. Strength (quadriceps) 3. Functional ability (30s-CST)	1 months 3 months	Not reported
Tungtrongjit et al. (17)	Strength training (3 times a day; 3 weeks)	Usual care	1. Pain (WOMAC) 2. Knee function (WOMAC) 3. Strength (quadriceps) 4. ROM 5. Stiffness (WOMAC) 6. WOMAC (total)	1 months 3 months 6 months	1 surgical wound infections, 1 post-operative knee trauma, 2 post-operative wound dehiscen (It was not stated whether adverse events occurred in the experimental group or the control group).
Skoffer et al. (18)	Strength training (3 times a week; 4 weeks)	Usual care	1. Pain (KOOS) 2. Knee function (KOOS) 3. Strength (quadriceps) 4. ROM 5. Functional ability (30s-CST)	1 weeks 3 months 12 months	None
Calatayud et al. (19)	Strength training (3 times a week; 8 weeks)	Usual care	1. Pain (VAS) 2. Knee function (WOMAC) 3. Strength (quadriceps) 4. ROM 5. Stiffness (WOMAC) 6. Physical function (SF-36) 7. WOMAC (total) 8. Functional ability (stair test)	1 months 3 months	Three patients in the control group had postoperative complications.
Jahic et al. (20)	Strength training (3 times a day; 6 weeks)	Usual care	1. Knee function (KS) 2. Physical function (FS)	After surgery 3 months 6 months 12 months	Not reported
Domínguez et al. (21)	Strength training (3 times a week; 4 weeks)	Usual care	1. Pain (KOOS) 2. Knee function (KOOS) 3. Strength (quadriceps) 4. ROM 5. Functional ability (TUG)	2 weeks 12 months	None
Leeuwen et al. (22)	Strength training (3 times a week; 6 weeks)	Usual care	1. Strength (quadriceps) 2. Functional ability (stair test) 3. WOMAC (total)	3 months	Not reported

*WOMAC, the Western Ontario and McMasters University Osteoarthritis Index; KOOS, Knee Injury and Osteoarthritis Outcome Score (KOOS); VAS, Visual Analog Scale; KS, Knee Score; FS, Function score; ROM, Range of Motion; SF-36, The MOS 36-item Short-Form Health Survey; TUG, Timed-Up-and-Go; 30s-CST, 30-second chair stand test.*

### Risk of Bias

**Figure 2:**
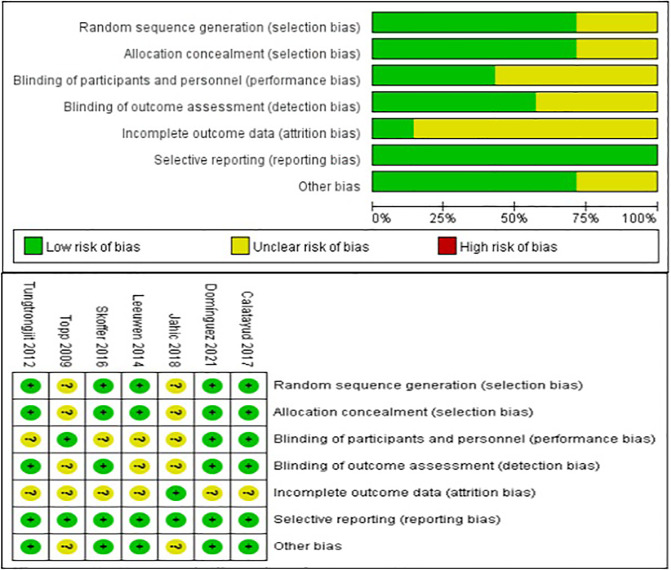
Risk of bias graph.

Figure 2 shows the risk of bias based on the seven RCTs assessed by the Cochrane Handbook. Five studies reported specific randomization methods (17–19, 21, 22), and two studies did not report specific randomization methods (16, 20). The allocation concealment of two studies was unclear(16, 20), and five studies were rated as low-risk (17–19, 21, 22). Three studies reported blinding of participants and personnel (16, 19, 21), and the other four studies did not specify this (17, 18, 20, 22). Four studies reported blinding of outcome assessment (17–19, 21), and the other three studies did not specify this (16, 20, 22). The attrition bias of six studies was rated as unclear because of the dropout rate (16–19, 21, 22), and the attrition bias of the other study was rated as low risk (20). The reporting bias of seven studies was rated as low risk (16–22), and the other bias of the two studies was rated as unclear (16, 22).

### Quality of Evidence

The overall quality of evidence for each outcome was assessed based on the GRADE approach. The results showed very low to moderate evidence in pain, strength (quadriceps), and functional ability, very low to low evidence in knee function, stiffness, WOMAC (total), and physical function, and low to moderate evidence in ROM and functional ability. Detailed results can be found in the Supplementary Appendix.

### Meta-Analysis

#### Pain

**Figure 3:**
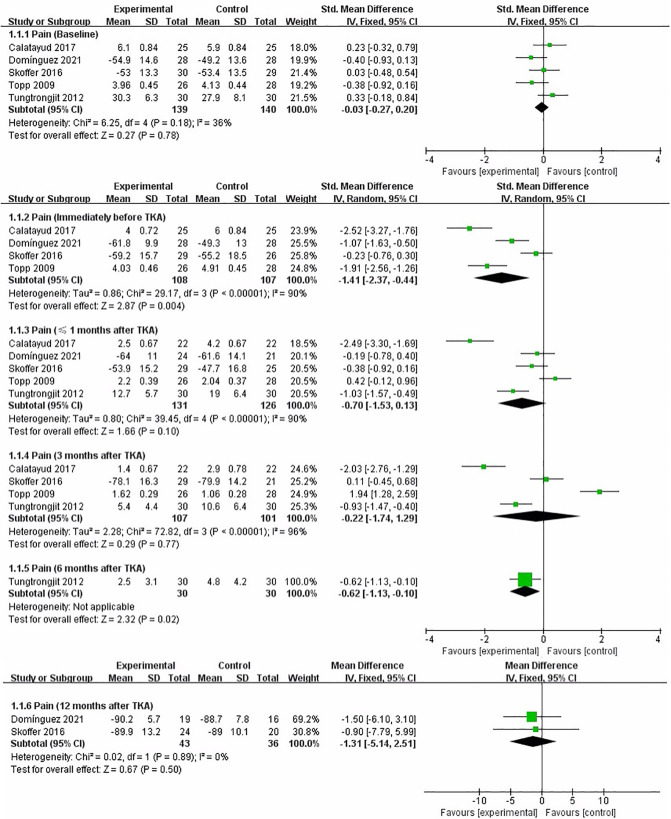
Meta-analysis on Pain.

We performed a meta-analysis to assess the effect of preoperative strength training on pain immediately before TKA, less than 1 month, 3, 6, and 12 months after TKA. Five studies that assessed pain used VAS (16, 19), KOOS (18, 21), and WOMAC subscales (17), respectively. At baseline assessment, the pooled results showed no significant difference in pain between strength training and control groups [SMD = −0.03, 95% CI (−0.27, 0.20), *I*^2^ = 36%, *P* = 0.78]. Immediately before TKA, the pooled results showed a significant improvement in pain in the strength training compared to the control group [SMD = −1.41, 95% CI (−2.37, −0.44), *I*^2^ = 90%, *P* = 0.004]. Overall, from the meta-analysis results, preoperative strength training has no significant effect on postoperative pain after TKA. Compared with the control group, the results indicated strength training had no statistically significant improvement in postoperative pain at less than 1 month [SMD = −0.70, 95% CI (−1.53, 0.13), *I*^2^ = 90%, *P* = 0.10], 3 month [SMD = −0.22, 95% CI (−1.74, 1.29), *I*^2^ = 96%, *P* = 0.77], and 12 month [MD = −1.31, 95% CI (−5.14, 2.51), *I*^2^ = 0%, *P* = 0.50]. However, the results indicated strength training had a statistically significant improvement in post-operative knee function at 6 month [SMD = −0.62, 95% CI (−1.13, 0.10), *P* = 0.02] (Figure 3).

### Knee Function

**Figure 4:**
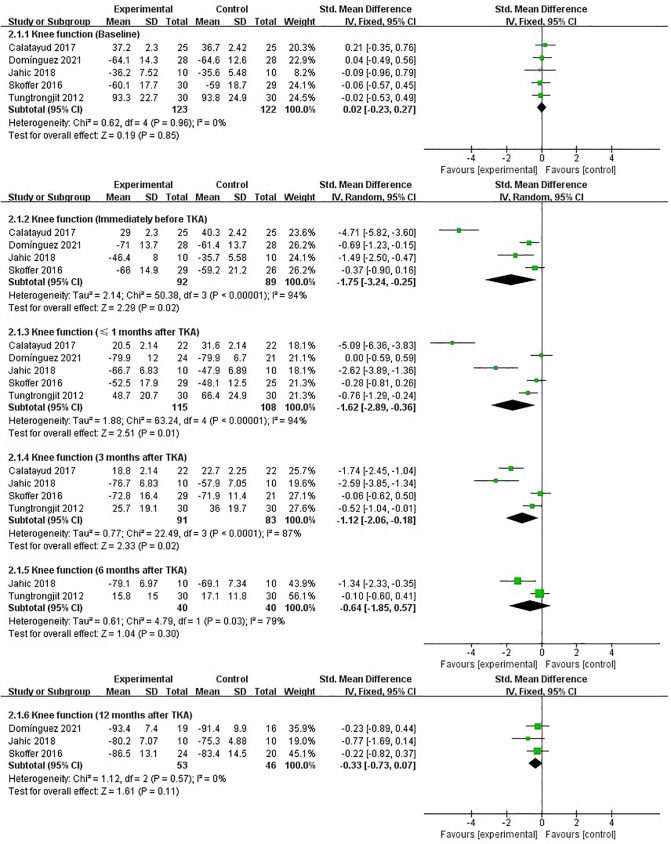
Meta-analysis on Knee function.

We performed a meta-analysis to assess the effect of preoperative strength training on knee function immediately before TKA, less than 1 month, 3, 6, and 12 months after TKA. Five studies that assessed knee function used KOOS (18, 21), WOMAC subscales (17, 19), and KS (20), respectively. At baseline assessment, the pooled results showed no significant difference in knee function between strength training and control groups [SMD = 0.02, 95% CI (−0.23, 0.27), *I*^2^ = 0%, *P* = 0.85]. Immediately before TKA, the pooled results showed a significant improvement in knee function in the strength training compared to the control group [SMD = −1.75, 95% CI (−3.24, −0.25), *I*^2^ = 94%, *P* = 0.02]. Compared with the control group, the results indicated strength training had a statistically significant improvement in post-operative knee function at less than 1 month [SMD = −1.62, 95% CI (−2.89, −0.36), *I*^2^ = 94%, *P* = 0.01], and 3 month [SMD = −1.12, 95% CI (−2.06, −0.18), *I*^2^ = 87%, *P* = 0.02]. However, the results indicated strength training had no statistically significant improvement in postoperative knee function at 6 month [SMD = −0.64, 95% CI (−1.85, 0.57), *I*^2^ = 79%, *P* = 0.30], and 12 month [SMD = −0.33, 95% CI (−0.73, 0.07), *I*^2^ = 0%, *P* = 0.11] (Figure 4).

### Strength (Quadriceps)

**Figure 5:**
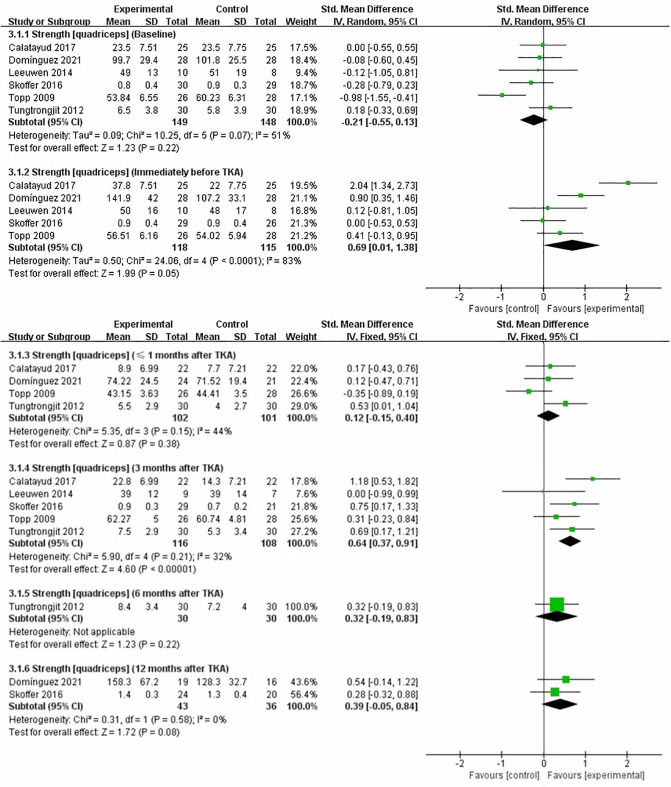
Meta-analysis on Strength (quadriceps).

We performed a meta-analysis to assess the effect of preoperative strength training on strength (quadriceps) immediately before TKA, less than 1 month, 3, 6, and 12 months after TKA. Six studies assessed strength (quadriceps) (16–19, 21, 22). At baseline assessment, the pooled results showed no significant difference in strength (quadriceps) between strength training and control groups [SMD = −0.21, 95% CI (−0.55, 0.13), *I*^2^ = 51%, *P* = 0.22]. Immediately before TKA, the pooled results showed no significant improvement in strength (quadriceps) in the strength training compared to the control group [SMD = 0.69, 95% CI (0.01, 1.38), *I*^2^ = 83%, *P* = 0.05]. Compared with the control group, the results indicated strength training had a statistically significant improvement in postoperative strength (quadriceps) at 3 month [SMD = 0.64, 95% CI (0.37, 0.91), *I*^2^ = 32%, *P* < 0.00001]. However, the results indicated strength training had no statistically significant improvement in postoperative strength (quadriceps) at less than 1 month [SMD = 0.12, 95% CI (−0.15, 0.40), *I*^2^ = 44%, *P* = 0.38], 6 month [SMD = 0.32, 95% CI (−0.19, 0.83), *P* = 0.22], and 12 month [SMD = 0.39, 95% CI (−0.05, 0.84), *I*^2^ = 0%, *P* = 0.08] (Figure 5).

### ROM

**Figure 6:**
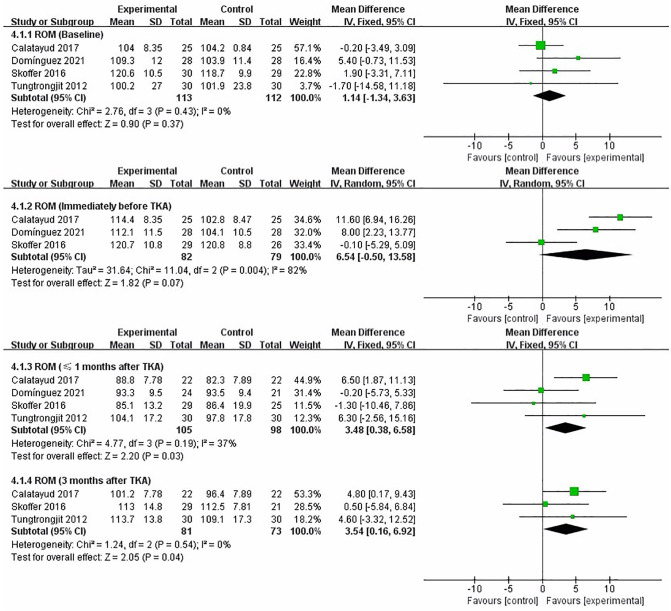
Meta-analysis on ROM.

We performed a meta-analysis to assess the effect of preoperative strength training on ROM immediately before TKA, less than 1 month and 3 months after TKA. Four studies assessed ROM (17–19, 21). The pooled results showed no significant difference in ROM between strength training and control groups at baseline assessment [MD = 1.14, 95% CI (−1.34, 3.63), *I*^2^ = 0%, *P* = 0.37]. Immediately before TKA, the pooled results showed no significant improvement in ROM in the strength training compared to the control group [MD = 6.54, 95% CI (−0.50, 13.58), *I*^2^ = 82%, *P* = 0.07]. From the meta-analysis results, preoperative strength training has a significant effect on postoperative ROM after TKA. Compared with the control group, the results indicated strength training had a statistically significant improvement in postoperative ROM at less than 1 month [MD = 3.48, 95% CI (0.38, 6.58), *I*^2^ = 37%, *P* = 0.03], and 3 month [MD = 3.54, 95% CI (0.16, 6.92), *I*^2^ = 0%, *P* = 0.04] (Figure 6).

### Functional Ability

**Figure 7:**
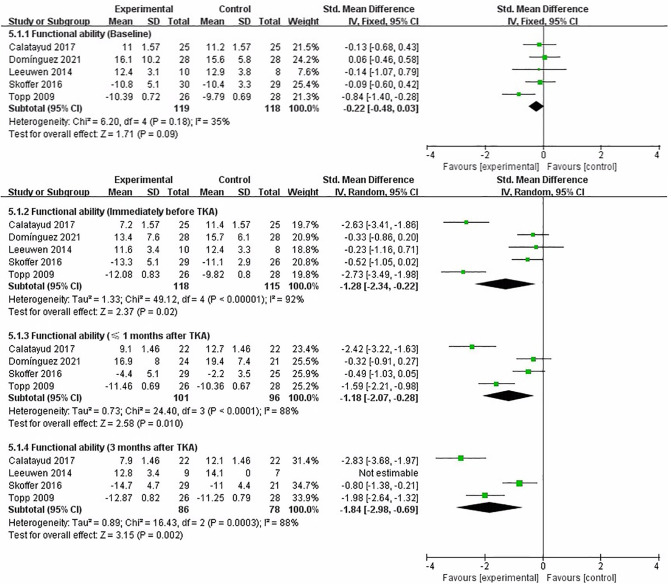
Meta-analysis on Functional ability.

We performed a meta-analysis to assess the effect of preoperative strength training on functional ability immediately before TKA, less than 1 month and 3 months after TKA. Five studies that assessed functional ability used stair test (19, 22), TUG (21), and 30s-CST (16, 18), respectively. The pooled results showed no significant difference in functional ability between strength training and control groups at baseline assessment [SMD = −0.22, 95% CI (−0.48, 0.03), *I*^2^ = 35%, *P* = 0.09]. Immediately before TKA, the pooled results showed a significant improvement in functional ability in the strength training compared to the control group [SMD = −1.28, 95% CI (−2.34, −0.22), *I*^2^ = 92%, *P* = 0.02]. From the meta-analysis results, preoperative strength training has a significant effect on postoperative functional ability after TKA. Compared with the control group, the results indicated strength training had a statistically significant improvement in postoperative functional ability at less than 1 month [SMD = −1.18, 95% CI (−2.07, −0.28), *I*^2^ = 88%, *P* = 0.01], and 3 month [SMD = −1.84, 95% CI (−2.98, −0.69), *I*^2^ = 88%, *P* = 0.002] (Figure 7).

### Stiffness

**Figure 8:**
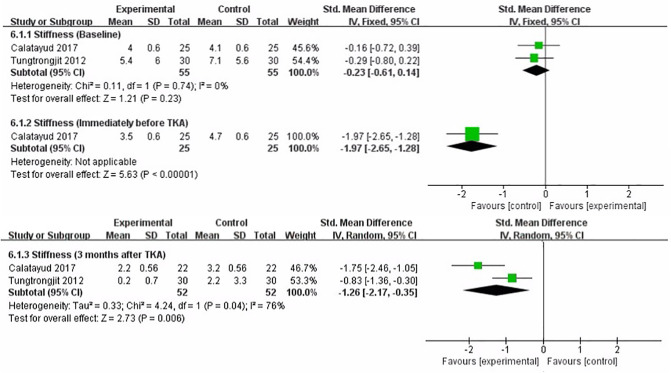
Meta-analysis on Stiffness.

We performed a meta-analysis to assess the effect of preoperative strength training on stiffness immediately before TKA, 3 months after TKA. Two studies reported stiffness using the WOMAC subscale (17, 19). The pooled results showed no significant difference in stiffness between strength training and control groups at baseline assessment [SMD = −0.23, 95% CI (−0.61, 0.14), *I*^2^ = 0%, *P* = 0.23]. Immediately before TKA, the pooled results showed a significant improvement in stiffness in the strength training compared to the control group [SMD = −1.97, 95% CI (−2.65, −1.28), *P* < 0.00001]. Compared with the control group, the results indicated strength training had a statistically significant improvement in postoperative stiffness at 3 month [SMD = −1.26, 95% CI (−2.17, −0.35), *I*^2^ = 76%, *P* = 0.006] (Figure 8).

### WOMAC (Total)

**Figure 9:**
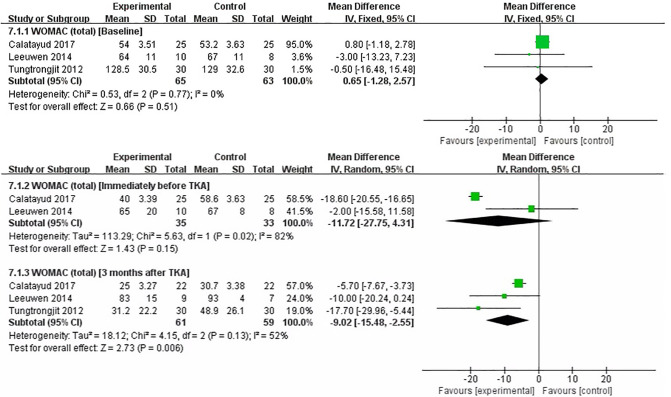
Meta-analysis on WOMAC (total).

We performed a meta-analysis to assess the effect of preoperative strength training on WOMAC (total) immediately before TKA, 3 months after TKA. Three studies assessed WOMAC(total) (17, 19, 22). The pooled results showed no significant difference in WOMAC (total) between strength training and control groups at baseline assessment [MD = 0.65, 95% CI (−1.28, 2.57), *I*^2^ = 0%, *P* = 0.51]. Immediately before TKA, the pooled results showed no significant improvement in WOMAC (total) in the strength training compared to the control group [MD = −11.72, 95% CI (−27.75, 4.31), *I*^2^ = 82%, *P* = 0.15]. Compared with the control group, the results indicated strength training had a statistically significant improvement in postoperative WOMAC (total) at 3 month [MD = −9.02, 95% CI (−15.48, −2.55), *I*^2^ = 52%, *P* = 0.006] (Figure 9).

### Physical Function

**Figure 10:**
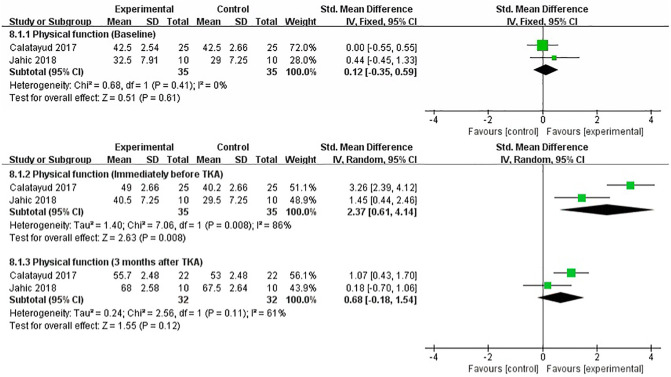
Meta-analysis on Physical function.

We performed a meta-analysis to assess the effect of preoperative strength training on physical function immediately before TKA, 3 months after TKA. Two studies that assessed physical function used SF-36 (19), and FS (20), respectively. The pooled results showed no significant difference in physical function between strength training and control groups at baseline assessment [SMD = 0.12, 95% CI (−0.35, 0.59), *I*^2^ = 0%, *P* = 0.61]. Immediately before TKA, the pooled results showed a significant improvement in physical function in the strength training compared to the control group [SMD = 2.37, 95% CI (0.61, 4.14), *I*^2^ = 86%, *P* = 0.008]. Compared with the control group, the results indicated strength training had no statistically significant improvement in postoperative physical function at 3 month [SMD = 0.68, 95% CI (−0.18, 1.54), *I*^2^ = 61%, *P* = 0.12] (Figure 10).

### Publication Bias

When the pooled results of the meta-analysis include more than ten studies, the potential publication bias should be reported (28). For continuous variables, Egger's and Begg's tests should be used to assess potential publication bias (29). Although none of the results included more than ten studies, we still used Egger’s and Begg's tests to assess publication bias. The specific results of the publication bias can be found in the Supplementary Appendix.

### Sensitivity Analysis

We used the leave-one-out method to assess the results with heterogeneity higher than 50%, including pain (immediately before TKA, less than 1 month, and 3 months after TKA), knee function (immediately before TKA, less than 1 month, 3 months, and 6 months after TKA), strength (quadriceps) (baseline, and immediately before TKA), ROM (immediately before TKA), functional ability (immediately before TKA, less than 1 month, and 3 months after TKA), stiffness (3 months after TKA), WOMAC(total) (immediately before TKA, and 3 months after TKA), and physical function (immediately before TKA, 3 months after TKA). Through sensitivity analysis, we found some potential sources of heterogeneity. In addition, after the assessments of the leave-one-out method, we found that the research results were generally robust. The specific results of the sensitivity analysis can be found in the Supplementary Appendix.

## Discussion

Quadriceps strength is closely related to knee function after TKA, and preoperative quadriceps weakness may lead to postoperative deterioration of TKA (30, 31). Previous studies have shown that surgery can damage the knee extension mechanism and the muscles around the knee joint (32). Therefore, improving muscle strength is of great significance for rehabilitation after TKA. Strength training is the most effective method to improve muscle strength, widely used in treating knee osteoarthritis and rehabilitation after TKA (33, 34). Although the previous meta-analysis assessed the efficacy of preoperative rehabilitation training for rehabilitation after TKA, the intervention of preoperative rehabilitation training was multimodal (35–39). Multimodal interventions may dilute the effect of individual factors or components of interventions, and it is difficult to assess their actual efficacy (40). However, our meta-analysis included studies with quadriceps exercise as the primary purpose, which may have reduced the mixed effects of the intervention in some ways. Second, these meta-analyses only assessed efficacy at a single time point, not at different times before and after surgery. In the current study, we evaluated the rehabilitation effects of preoperative strength training at different time points before and after TKA, and the outcomes included pain, knee function, strength (quadriceps), ROM, functional ability, stiffness, WOMAC (total), and physical function. Additionally, some studies were not limited in terms of surgical approach. The effect of different surgical methods on postoperative recovery and intervention may be inconsistent. Our study limited the surgical approach. The surgical approach for TKA is limited to an anterior midline skin incision and a medial parapatellar approach. A consistent surgical approach may contribute to clinical decision-making for TKA. In similar studies that have been published previously (35–39), two studies found no significant advantage of preoperative training on postoperative clinical outcomes (37, 38). Chen et al. found that preoperative training could effectively shorten the length of hospital stay but had no significant effect on clinical outcomes (36). Liu et al. found that preoperative training improved physical function in patients undergoing TKA (39). In addition, Wang et al. found that preoperative rehabilitation may slightly improve early postoperative pain and function in patients with TKA but has no effect on key outcomes. However, this effect is so small that its clinical significance may be considered insignificant (35). Our study also found that preoperative strength training showed advantages for early postoperative clinical outcomes. However, due to limitations in the included literature, we did not assess some other outcomes, such as length of hospital stay and medical costs. The clinical significance of the present findings may also be considered insignificant.

We found that patients undergoing TKA who received preoperative strength training showed better clinical efficacy in many aspects compared with the control group. Patients who received preoperative strength training showed better pain, knee function, functional ability, stiffness, and physical function immediately before TKA. However, strength training did not show significant advantages in improving strength (quadriceps), ROM, and WOMAC (total) immediately before TKA. Patients who received preoperative strength training showed better knee function, ROM, and functional ability at less than 1 month and 3 months after TKA. However, strength training did not show significant advantages in improving knee function at 6 and 12 months after TKA. In addition, patients who received preoperative strength training showed better strength (quadriceps), stiffness, and WOMAC (total) at 3 months after TKA. However, preoperative strength training did not show significant advantages in improving strength (quadriceps) at less than 1 month, 6, and 12 months after TKA. These may be related to surgical injury and other postoperative complications because the healing time of normal tissues usually takes 3 months (41). Preoperative strength training showed no significant advantage in improving strength (quadriceps) at less than 1 month, 6, and 12 months, which may be related to the long-term efficacy of strength training. Muscle strength is closely related to knee function, and improving muscle strength can improve ROM and knee function (30, 36). In addition, functional ability may be affected by knee function and muscle strength. Due to the study's limitations, the included RCT did not report the long-term follow-up data of ROM, functional ability, and WOMAC (total). In general, our study found that the benefits of preoperative strength training in improving ROM, functional ability, knee function, strength (quadriceps), and WOMAC (total) were mainly concentrated in the early postoperative period, but no long-term efficacy was found, which requires further verification by more RCT.

Postoperative pain is one of the most important complications after TKA, which seriously affects the patient's quality of life (42). The mechanism of postoperative pain is very complicated and affected by many factors (41). Overall, our study found no advantage of preoperative strength training in improving pain after TKA, which may be related to the trauma caused by the surgery. In addition, the pooled results showed that strength training had a significant advantage in improving stiffness after TKA but not in improving physical function. Postoperative stiffness is related to surgical factors, such as surgical technique, surgical trauma, and pain (43). The physical function of patients after TKA is also affected by many aspects, such as psychological state and physical activity in the early postoperative period (44, 45). However, since there are only two studies evaluating stiffness and physical function and the number of patients is also small, the clinical significance of the pooled results needs to be further verified by more studies.

This study assessed data for all outcomes at baseline and immediately before TKA. In addition, this study assessed clinical outcomes at different time points after TKA. However, the included literature had some limitations, and some studies had no long-term follow-up. Therefore, this study assessed the effects of strength training on pain, knee function, and strength (quadriceps) at less than 1 month, 3, 6, and 12 months after surgery, ROM and functional ability at less than 1 month and 3 months after surgery, and WOMAC (Total), stiffness, and physical function at 3 months after surgery. The implementation of RCTs related to TKA has great challenges, and some studies lack long-term follow-up data, and further research is needed in the future to draw more comprehensive results and conclusions. Although this study found that preoperative strength training showed an advantage in the early clinical results after TKA, considering that this effect is too small, the long-term evaluation does not show an advantage, and some other vital results (length of hospital stay and medical expenses) have not been assessed, the clinical significance of the results may need to be interpreted more carefully.

We had registered the protocol with PROSPERO before the study started. Due to literature limitations, there are some differences between our manuscript and the pre-registered protocol. First of all, the retrieval time in the pre-registration protocol is until July 1, 2021. Due to the busy work, we will postpone the retrieval time to November 17, 2021. This change did not affect our final results. Second, the pre-registered protocol did not limit the surgical approach. For consistency, we have restricted the surgical approach. Third, the pre-registered protocol does not include the result of WOMAC (total). Considering the importance of this result, we added the content of this result to our study. Overall, the study was conducted according to a pre-registered protocol. These slight changes did not have a significant impact on the entire study.

### Limitations

There were several limitations of this study that should be considered. Firstly, there are some methodological limitations in the included studies, such as the blinding of participants and personnel, the blinding of outcome assessment, and the dropout rate, which may have a potential impact on the results. Secondly, the follow-up time of the included study was short. This study assessed the efficacy of pain, knee function, and strength (quadriceps) from 1 month to 12 months after surgery, while the other results could not be assessed for long-term efficacy due to the absence of long-term follow-up data. Thirdly, Although this study included literature with the primary purpose of quadriceps training, other muscles around the knee joint may be potentially trained during quadriceps training as they act synergistically. Therefore, it may be challenging to be sure that only the quadriceps are being trained. Fourthly, the number of included studies was small, and none of the results included more than ten studies, and there may be some bias in the study results. Fifthly, the number of included studies was small, and none of the results included more than ten studies, and there may be some bias in the study results. Fifthly, we excluded irrelevant literature by reading the title and abstract, and there is the possibility of missing some important literature. In addition, this study could not perform subgroup analyses based on intervention characteristics, such as duration of intervention and frequency of intervention, due to the small number of included studies. Finally, the total sample size of the included studies was small, and it is necessary to expand the sample size for future studies to draw more definitive conclusions.

### Implications for Further Research and Practice

Higher quality evidence is still needed before definitive conclusions can be drawn. We acknowledge that there are some difficulties in conducting this type of RCTs, but there are still ways to improve the study's quality further. First of all, future research should strictly follow the CONSORT guidelines to improve the quality of research (46). At the same time, stricter restrictions should be carried out in methodologies, such as randomization methods, rigorous blinding methods, and reduction of sample dropout rates. Second, research should extend the follow-up time and increase the frequency of follow-up to assess long-term efficacy in the future. Third, the safety of interventions is critical, and future studies should report adverse events more comprehensively to assess their safety.

## Conclusions

Preoperative strength training may be beneficial to early rehabilitation after TKA, but the long-term efficacy needs to be further determined. Given the limitations of this study, the conclusions of this study are preliminary, and we cannot draw firm conclusions based on the current findings. At the same time, more caution should be exercised when interpreting the clinical efficacy of preoperative strength training for TKA.

## Data Availability

The original contributions presented in the study are included in the article/**Suplementary Material**, further inquiries can be directed to the corresponding author/s.
