# Supplementary material for: Preoperative Strength Training for Clinical Outcomes Before and After Total Knee Arthroplasty: A Systematic Review and Meta-Analysis
Source: Front Surg. 2022 Jul 21;9:879593. doi: 10.3389/fsurg.2022.879593 (PMC9349363; doi:10.3389/fsurg.2022.879593)
Supplement: Supplementary file 1 [file Table_1_v1.docx]

**TABLE 1** Study characteristics.

| **References** | **Diagnosis** | **Type of operation** | **Publication years** | **Country** | **Study design** | **Mean age (SD), years** | | **Sample size** | | **Male/Female** | | **Droup out** | |
| --- | --- | --- | --- | --- | --- | --- | --- | --- | --- | --- | --- | --- | --- |
|  |  |  |  |  |  | **IG** | **CG** | **IG** | **CG** | **IG** | **CG** | **IG** | **CG** |
| Topp et al. (16) | KOA^a^ | TKA | 2009 | USA | RCT | 64.10(7.05) | 63.50(6.68) | 26 | 28 | 17/37 | | NR | |
| Tungtrongjit et al. (17) | KOA^a^ | TKA | 2012 | Thailand | RCT | 63.00(7.60) | 65.90(7.20) | 30 | 30 | 4/26 | 6/24 | 8 | |
| Skoffer et al. (18) | KOA^a^ | TKA | 2016 | Denmark | RCT | 70.70(7.30) | 70.10(6.40) | 30 | 29 | 11/19 | 12/17 | 6 | 9 |
| Calatayud et al. (19) | KOA^b^ | TKA | 2017 | Spain | RCT | 66.80(4.80) | 66.70(3.10) | 25 | 25 | 4/18 | 3/19 | 3 | 3 |
| Jahic et al. (20) | KOA^a^ | TKA | 2018 | Bosnia and Herzegovina | RCT | NR | | 10 | 10 | 3/7 | 3/7 | 0 | 0 |
| Domínguez et al. (21) | KOA^a^ | TKA | 2021 | Spain | RCT | 70.80(5.40) | 70.20(5.60) | 24 | 21 | 10/14 | 7/14 | 5 | 5 |
| Leeuwen et al. (22) | KOA^a^ | TKA | 2014 | Netherlands | RCT | 71.80(7.50) | 69.50(7.10) | 10 | 8 | 7/3 | 4/4 | 1 | 3 |

ACR, American College of Rheumatology; IG, Intervention group; CG, Control group; RCT, Randomized controlled trial; NR, Not reported; KOA^a^, physician diagnoses; KOA^b^, American College of Rheumatology (ACR) diagnostic criteria; TKA, Total knee arthroplasty;

**TABLE 2** Intervention Characteristics and Outcome Measures.

| **References** | **Intervention characteristics** | | **Main outcomes and results** | **Follow-up** | **Adverse events** |
| --- | --- | --- | --- | --- | --- |
|  | **Intervention group** | **Control group** |  |  |  |
| Topp et al. (16) | Strength training (3 times a week; 20weeks) | Usual care | 1. Pain (VAS)  2. Strength (quadriceps)  3. Functional ability (30s-CST) | 1 months  3 months | Not reported |
| Tungtrongjit et al. (17) | Strength training (3 times a day; 3weeks) | Usual care | 1. Pain (WOMAC)  2. Knee function (WOMAC)  3. Strength (quadriceps)  4. ROM  5. Stiffness (WOMAC)  6. WOMAC (total) | 1 months  3 months  6 months | 1 surgical wound infections, 1 post-operative knee trauma, 2 post-operative wound dehiscen (It was not stated whether adverse events occurred in the experimental group or the control group). |
| Skoffer et al. (18) | Strength training (3 times a week; 4weeks) | Usual care | 1. Pain (KOOS)  2. Knee function (KOOS)  3. Strength (quadriceps)  4. ROM  5. Functional ability (30s-CST) | 1 weeks  3 months  12 months | None |
| Calatayud et al. (19) | Strength training (3 times a week; 8weeks) | Usual care | 1. Pain (VAS)  2. Knee function (WOMAC)  3. Strength (quadriceps)  4. ROM  5. Stiffness (WOMAC)  6. Physical function (SF-36)  7. WOMAC (total)  8. Functional ability (stair test) | 1 months  3 months | Three patients in the control group had postoperative complications. |
| Jahic et al. (20) | Strength training (3 times a day; 6weeks) | Usual care | 1. Knee function (KS)  2. Physical function (FS) | After surgery  3 months  6 months  12 months | Not reported |
| Domínguez et al. (21) | Strength training (3 times a week; 4weeks) | Usual care | 1. Pain (KOOS)  2. Knee function (KOOS)  3. Strength (quadriceps)  4. ROM  5. Functional ability (TUG) | 2 weeks  12 months | None |
| Leeuwen et al. (22) | Strength training (3 times a week; 6weeks) | Usual care | 1. Strength (quadriceps)  2. Functional ability (stair test)  3. WOMAC (total) | 3 months | Not reported |

WOMAC, the Western Ontario and McMasters University Osteoarthritis Index; KOOS, Knee Injury and Osteoarthritis Outcome Score (KOOS); VAS, Visual Analog Scale; KS, Knee Score; FS, Function score; ROM, Range of Motion; SF-36, The MOS 36-item Short-Form Health Survey; TUG, Timed-Up-and-Go; 30s-CST, 30-second chair stand test;
